# Clinician and Patient Responses to US Health Insurers' Policies: A Qualitative Study of Higher Risk Patients

**DOI:** 10.1111/1475-6773.14615

**Published:** 2025-04-24

**Authors:** Tammy L. Eaton, Valerie Danesh, Abigail C. Jones, Christine C. Kimpel, Carla M. Sevin, Han Su, Kelly M. Toth, Thomas S. Valley, Theodore J. Iwashyna, Leanne M. Boehm, Joanne McPeake

**Affiliations:** ^1^ VA Center for Clinical Management Research, VA Ann Arbor Healthcare System Ann Arbor Michigan USA; ^2^ Department of Internal Medicine, Division of Hospital Medicine Michigan Medicine Ann Arbor Michigan USA; ^3^ Institute for Healthcare Policy and Innovation, University of Michigan Ann Arbor Michigan USA; ^4^ Center for Applied Health Research Baylor Scott & White Health Dallas Texas USA; ^5^ Department of Medicine Baylor College of Medicine Houston Texas USA; ^6^ School of Nursing Vanderbilt University Nashville Tennessee USA; ^7^ Department of Medicine, Division of Allergy, Pulmonary, and Critical Care Medicine Vanderbilt University Medical Center Nashville Tennessee USA; ^8^ CRISMA Center, Critical Care Medicine University of Pittsburgh Pittsburgh Pennsylvania USA; ^9^ Department of Internal Medicine, Division of Pulmonary and Critical Care Medicine University of Michigan Ann Arbor Michigan USA; ^10^ VA Center for Clinical Management Research Ann Arbor Michigan USA; ^11^ Center for Bioethics and Social Sciences in Medicine University of Michigan Ann Arbor Michigan USA; ^12^ Division of Pulmonary & Critical Care Medicine, School of Medicine Johns Hopkins Bloomberg School of Public Health Baltimore Maryland USA; ^13^ Department of Health Policy and Management Johns Hopkins University Baltimore Maryland USA; ^14^ Critical Illness, Brain Dysfunction, and Survivorship (CIBS) Center Vanderbilt University, Medical Center Nashville Tennessee USA; ^15^ The Healthcare Improvement Studies Institute University of Cambridge Cambridge UK

**Keywords:** access to care, adverse events, financial hardship, health insurance, ICU recovery, post‐ICU clinic, workaround

## Abstract

**Objective:**

To identify specific ways in which US health insurance triggered changes in care and interrupted the encounter between clinicians and patients in post‐intensive care unit (ICU) clinics.

**Study Setting and Design:**

This naturalistic qualitative study was nested within a randomized controlled trial that evaluated the feasibility and preliminary efficacy of a telemedicine ICU recovery clinic intervention. Adult participants were referred to a multidisciplinary ICU recovery clinic after septic shock or acute respiratory distress syndrome (ARDS) in a Southeastern US academic medical center.

**Data Sources and Analytic Sample:**

Data were collected from 2019 to 2021. Telemedicine ICU recovery visits within the intervention group were used in this analysis. ICU recovery visits at 3‐ and 12‐week intervals after hospital discharge were recorded and analyzed based upon the constant comparative method. Responses were initially open coded and then consolidated with the Donabedian Model of assessing healthcare quality by two investigators to organize themes and subthemes, with discrepancies in coding resolved by consensus.

**Principal Findings:**

Thirty‐three clinic visit transcripts from 19 patients revealed health insurance‐related issues commonly elicited by clinicians. One in three patients raised health insurance‐related issues during their clinical encounter. Structural barriers to ICU recovery included high out‐of‐pocket spending, the complexity of interfacing with health insurance companies, and health insurance literacy. Patients initiated modifications to intended care to overcome insurance‐related barriers to recovery, including nonadherence to prescribed medications and treatments and crafting unsafe “workarounds” to recommended healthcare, with consequences to their recovery.

**Conclusions:**

We found that health insurance complexity and high out‐of‐pocket costs compromise the quality of care and recovery experienced by ICU survivors. These findings emphasize the need for solutions at the policy, payor, and healthcare system levels to mitigate barriers to ICU recovery created by health insurance, which can adversely influence affordable, timely, and appropriate critical illness survivor care.

**Trial Registration:** NCT03926533

**Summary**

- What is known on this topic:
  ○ ICU survivors commonly experience socioeconomic distress tied to health care access and disability, potentially exacerbated by the complexity of the US health insurance system.
- What this study adds:
  ○ Findings of this study provide initial insights into the barriers associated with obtaining and using US health insurance to access critical illness survivor care. These barriers may adversely influence affordable, timely, and appropriate follow‐up care.

## 1 Introduction

US health insurance companies seek to improve the efficiency and quality of care by shaping multiple features of clinical encounters. The rationale for adding this additional complexity and layer of management is that it will result in improved clinical decision‐making and outcomes by steering care. However, such policies are often guided by evidence toward average patients and may be a poor fit for patients with significant complexity or unusual experiences.

The millions of Americans surviving critical illness each year may be such a population at high risk for unintended consequences of insurance policies, as these patients experience considerable long‐term problems (e.g., new or worsening physical, cognitive, psychological impairment), with subsequent socioeconomic distress tied to accessing ongoing care and disability resulting in loss of employment [1, 2, 3] and personal and household financial burden, and time off from work [4, 5, 6]. Insurance company policies may become a concentrating lens, bringing together physiologic, socioeconomic, literacy [7], and administrative challenges. Yet despite the fact that interactions with insurance companies are often the first thing that occurs at every clinic visit (as updated insurance information is taken at check‐in) and the last thing that occurs at every visit (as a co‐pay is taken), there has been little sustained study of the ways in which insurance company policies shape the visits themselves.

We sought to understand how insurance company policies shape the processes of care delivered to ICU survivors as a population at high risk for making unintended and/or maladaptive responses. Existing studies focused on inpatient care demonstrate that such shaping occurs through mechanisms other than just expenses [8, 9, 10]. Therefore, we identified specific ways in which health insurance led to changes in care and shaped the dialogue between clinicians and patients in post‐ICU clinics.

## 2 Methods

This naturalistic qualitative study was nested within a randomized controlled trial (RCT) evaluating the feasibility and efficacy of a telemedicine ICU recovery clinic intervention (NCT03926533). The multidisciplinary ICU recovery clinic is located within the pulmonary clinic of a Southeastern United States academic medical center [11]. The study was approved by the Vanderbilt University Institutional Review Board (#190790) and is reported according to the Consolidated Criteria for Reporting Qualitative Research (COREQ) (Data S1).

### 2.1 Sampling and Recruitment

Adults (18 years and older) hospitalized for septic shock or acute respiratory distress syndrome (ARDS) were consecutively screened for eligibility [11]. Exclusion criteria included out of state residence; hospice referral; acute neurologic injury; or preexisting severe substance abuse, psychiatric disorder, or dementia. Participants were also excluded if connectivity for a telemedicine visit was not possible. Those who declined enrollment in the RCT were offered usual care via a referral to the ICU recovery clinic, if indicated. Patients who met inclusion criteria were approached for written consent. Participants were randomized 1:1 to receive telemedicine follow‐up (intervention) or usual care as designated by the discharging clinical team.

### 2.2 Data Collection

Telemedicine ICU recovery visits within the intervention group were used in this analysis. These 1‐h visits occurred at approximately 3 and 12 weeks after hospital discharge. Participants met with the clinic's ICU physician, ICU pharmacist, and psychologist via a secure web‐conferencing platform. Patient‐centered clinical assessments performed during each visit to address new or worsening cognitive, physical, and/or mental health concerns have been reported previously [11]. As part of the structured clinical assessment, recorded in the electronic health record, the clinic's pharmacist was prompted to ask, “if the cost of medications is a problem.” These clinicians were the same individuals for all study visits. The naturalistic dialogue represents field observations without any manipulation or interference, thus offering ecological validity for applicability and representativeness of real‐world settings.

Baseline demographics were collected from all participants at enrollment. During the 24‐month study period (2019–2021), 33 ICU recovery clinic visits were audio recorded and included in the analysis, with no participant refusals to record. Audio recordings were transcribed verbatim using an IRB‐approved transcription service and de‐identified for analysis.

### 2.3 Qualitative Data Analysis and Rigor

Data were analyzed to explore ways in which health insurance influenced changes in care and interrupted the dialogue between clinicians and patients. During visits, patients were asked about insurance provisions and the affordability of medicines as part of clinical care.

Analysis was based upon the constant comparative method [12]. “Open” codes which described each phenomenon found were initially generated. Through comparison across transcripts, relationships between the open codes were then explored, refined, and organized to showcase their relationships. We then applied the Donabedian Model of assessing healthcare quality [13] as the guiding framework to identify trends and patterns within each component, exploring how structural elements influence processes and ultimately impact patient outcomes. Donabedian suggests that high‐quality healthcare depends on having appropriate structures in place, providing effective and efficient processes of care, and achieving positive health outcomes [14].

Specifically, five transcripts were open coded by two researchers (J.M. and T.L.E.) to develop an initial coding frame, which was applied to subsequent transcripts. This continued until all transcripts were analyzed and no new codes were added to the coding frame. All transcripts were coded once, with intermittent double coding (20% of transcripts) to avoid idiosyncratic coding. Differences were discussed and remaining discrepancies were resolved via consensus. Key quotes to support findings were extracted. An audit trail, analytic memos, and peer debriefings were used to guard against bias. Multiple investigators representing different settings and specialties participated in iterative analysis and writing, thus enhancing the credibility.

## 3 Results

Nineteen participants requiring ICU admission for septic shock and/or ARDS completed 33 telemedicine ICU recovery clinic visits at either 3 weeks posthospital discharge (16%), 12 weeks posthospital discharge (5%), or both (79%) (Table 1). Prehospital baseline insurance coverage status and patient self‐reported health disparities can be found in Table 2. Family caregivers participated in 13 telemedicine ICU recovery clinic visits. Thirteen visits (39%) included health insurance‐related issues, seven (41%) occurring during the 3‐week visit, and six (38%) occurring during the 12‐week visit. Six participants (32%) reported health insurance‐related issues at both visits. During the initial 3‐week visit, over one‐third of patients initiated discussion surrounding insurance‐related issues before and/or apart from the cost of medications being clinically assessed.

**TABLE 1:** Characteristics of participants (*n* = 19).

Variable	Value
Age, years, median (IQR)	49.1 ± 16.3
Female, *n* (%)	10 (53)
Self‐reported race and ethnicity, *n* (%)	
Black	2 (11)
Hispanic	1 (5)
White	17 (89)
Diagnosis^a^	
Septic shock	18 (95)
ARDS	3 (16)
Mechanical ventilation (*n* = 8), days	3 (2–6.5)
ICU days	3 (2–9)
Hospital days	16 (12–22)
Discharge destination	
Home	15 (79)
Inpatient rehabilitation, skilled nursing facility, or long‐term acute care hospital	4 (21)
Telehealth visits with health insurance‐related issues	
3‐week visit (*n* = 17)	7 (41)
12‐week visit (*n* = 16)	6 (38)
Participants with health insurance‐related issues at both visits	6 (32)
Patient‐reported health insurance coverage status during follow‐up, *n* (%)	
3‐week visit (*n* = 17)	
Yes	13 (76)
No	1 (6)
Pending	1 (6)
Not reported	2 (12)
12‐week visit (*n* = 16)	
Yes	14 (88)
No	1 (6)
Not reported	1 (6)

*Note:* Data are presented as no. (%), mean ± standard deviation, or median (interquartile range).

Abbreviations: ARDS = acute respiratory distress syndrome; IQR = Interquartile range; ICU = intensive care unit.

^a^ Some participants experienced both septic shock and ARDS.

**TABLE 2:** Prehospital insurance status and self‐report of health disparities (*N* = 19).

Variable	Value, *n* (%)
Prehospital insurance coverage	
Private insurance	10 (53)
Medicare/Medicare managed plan	8 (42)
Medicaid	1 (5)
In the past year, have you or any family members you live with been unable to get any of the following when it was really needed?	
Food	0
Utilities	0
Medicine or any health‐care	0
Phone	0
Clothing	0
Child care	0
How hard is it for you to pay for the very basics like food, housing, medical care, and heating?	
Very hard	1 (5)
Somewhat hard	3 (16)
Not hard at all	15 (79)
Has lack of transportation kept you from medical appointments, meetings, work, or from getting things needed for daily living?	
Yes, it has kept me from medical appointments or from getting my medications	3 (16)
Yes, it has kept me from non‐medical meetings, appointments, work or things I need	1 (5)
No	16 (84)

**FIGURE 1:**
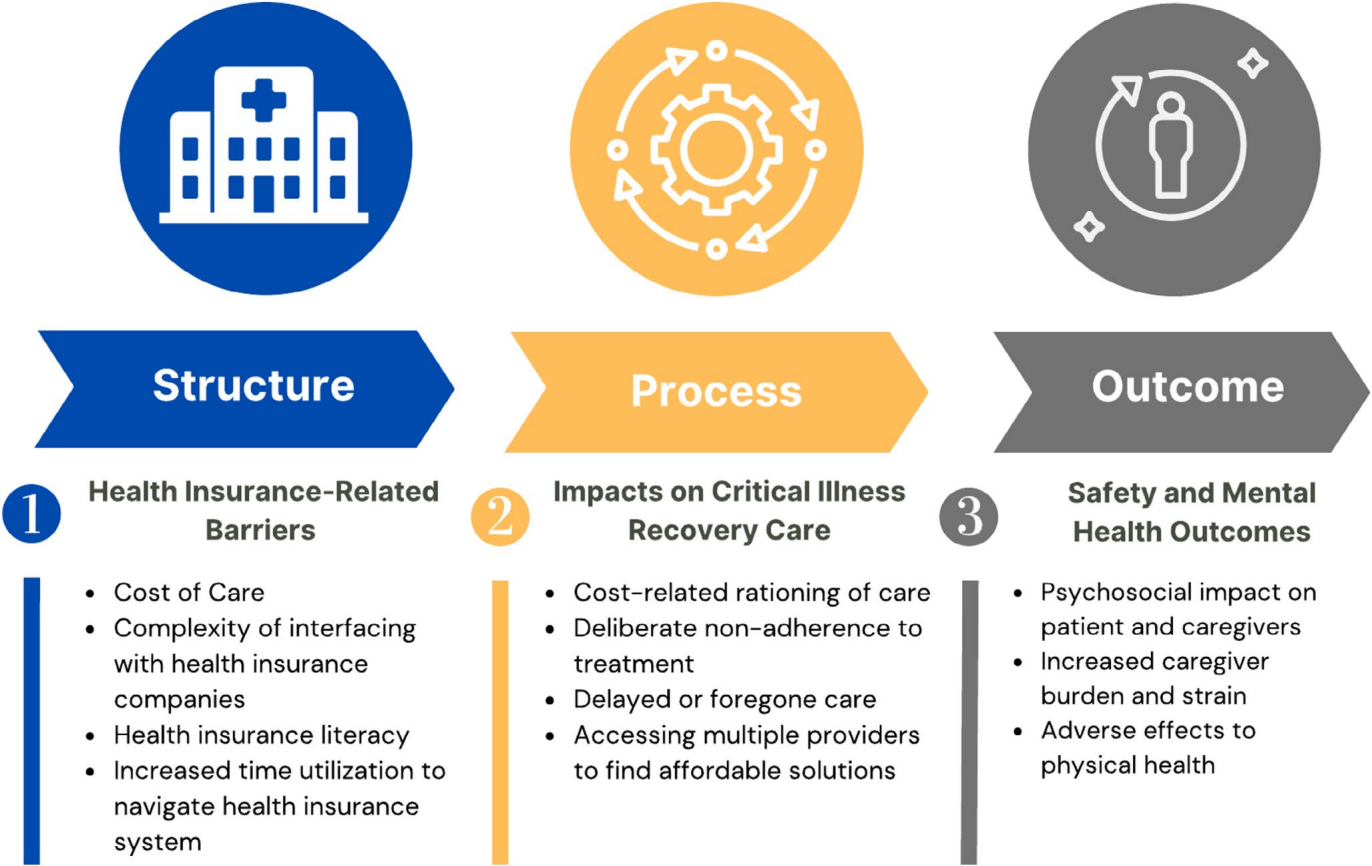
Themes and subthemes categorized using the Donabedian framework of structure‐process‐outcome.

The findings are organized within the Donabedian structure, process, and outcome model constructs, with three major themes emerging: (1) *Structure:* patient, caregiver, and/or clinician identified health insurance barriers interfering with access to prescribed care; (2) *Process:* changes in prescribed critical illness recovery care; and (3) *Outcome:* impact on safety and psychosocial outcomes of survivors of critical illness and their caregivers (Figure 1). A detailed explanation of each theme is presented, alongside representative excerpts.

### 3.1 Structure Dimension: Health Insurance Barriers

Several structural health insurance‐related barriers were discovered during the visits. These include the cost of care as it relates to out‐of‐pocket spending; the complexity of interfacing with health insurance companies; and health insurance literacy and its impact on accessing safe care.

Participants identified the cost of care, especially out‐of‐pocket expenses for medications, as a key barrier to recovery. One participant and their family caregiver discussed their inability to obtain prescribed antifungals and antihypertensives due to changes in insurance coverage:

Participant #18, Visit 1

Patient “Right now, my insurance is pending, I haven't been able to refill the [antifungal medication] and I forgot which other one.”

Caregiver “The metoprolol…the heart one.”

Patient “Yeah, the heart one, because I'm waiting for my insurance to go through, it's pending right now, so I can't refill those.”

Participants also described the complexity and time expense when interfacing with health insurance companies. Insurance prior authorization requirements reportedly slowed efforts to fill an essential analgesic medication prescription. The participant initiated communication with the insurance company, pharmacy, and physician's office, and ultimately paid out‐of‐pocket expenses despite having health insurance that covered the medication.

Participant #27, Visit 1

Patient “[Health insurance company representative] just talked to the pharmacy. They say they ‘are going to need a new [pain medication] prescription from your doctor.’ … I call the pharmacy and they are like… ‘yep’, I call the doctor, they go ‘nope, it should be fine, we sent a new script [prescription]’.‘I call the pharmacy again,’ ‘…there's a difference between [short acting pain medication] and [long‐acting pain medication] and we think the insurance company has written the wrong prior authorization…you could get it out of pocket?’. So, I dropped a few hundred bucks…I was so sick and tired…I can't keep doing this. I can't keep being the man in the middle.”

In this cohort we noted that health insurer websites placed demands on patients that surpassed their health insurance literacy. One participant reported waiting for inpatient hospital staff to connect them with a primary care provider (PCP) and had not seen a PCP within the first 3 months after hospital discharge because the website design exceeded what they could manage independently.

Participant #18, Visit 2

Patient “And then the lady from [hospital inpatient staff], I forgot her name, she was supposed to help me find somebody in [city] since I have my insurance now, so I wouldn't have to go to different places, but they still haven't referred me to a new PCP yet.”

ICU Physician “I'm not sure, there are some insurance restrictions in terms of getting a PCP. You're here in [city]?”Patient

“Yes.”

ICU Physician “Many insurance websites, they'll have a list online of the primary care doctors that take that insurance, so that might be worth checking out just to start.”Patient

“Okay. You said on the insurance, like the website?”

ICU Physician “Yeah. If you go to [insurance carrier], they should have a website with doctors that are in the plan.”

### 3.2 Process Dimension: Impact of Health Insurance Barriers on Prescribed Critical Illness Recovery Care

Participants described implementing processes and “workarounds” for barriers caused by health insurance. Participants repeatedly reported rationing and deliberate nonadherence to prescribed medications (e.g., anticoagulants, insulin) due to financial constraints. For example, one participant did not adhere to their insulin prescription due to affordability.

Participant #8, Visit 1

ICU Physician “…speaking of your blood clot, you're not on blood thinners right now because you weren't able to get the [anticoagulant]?”

Patient “When I left the hospital, they said it would be $141… and it's just a really hard time right now, I sure couldn't do that right then. But I got what the insurance would pay for. And then my [commercial pharmacy] told me it was ready after I left the hospital, but I didn't go get it. Because I knew it was going to be that amount.”

ICU Physician “So how many days have you been without that medicine?”

Patient “Probably about three.”

ICU Physician “Do you have a plan to get it now?”

Patient “Yeah. I just called them today. I got a thing in the mail yesterday with the program that'll help me out with it.”

Another participant ordered a prescription medication from an international supplier via an unverified website, recommended by their physician.

Participant #23, Visit 1

Patient “This is the one that's so expensive [medication]. Fifty dollars a pill. That's one I'm glad to find an alternative though.”

ICU Pharmacist “Well, unfortunately there's not one. I think that's why it's so expensive, because there's only one on the market.”

Patient “I ordered from a Canadian pharmacy where they're only a dollar.”

ICU Pharmacist “You have to be careful because sometimes when you order stuff like that it might not work, that makes me question if it's the real thing.”

Patient “This is a pharmacy that was given to me by my personal physician. He says he's used it a lot with patients.”

There were other ways in which insurance requirements changed the care patients received. Valuable time during clinic visits was spent discussing ways to decrease out‐of‐pocket expenses of care.

Participant #34, Visit 1

ICU physician “Since you've been home, do you have any home services?”

Family caregiver “No, we've not used any home‐health and I'm actually a nurse and yes, I have worked for an orthopedic group in [city]. So, we have been doing physical therapy through our [caregiver employer] outpatient therapy location. So that way we don't have to pay a deductible for the visits.”

ICU recovery clinic visits had to develop approaches to helping patients access care that was affordable and accessible. This included accessing multiple pharmacies in various locations across the city:

Participant #18, Visit 1

Family caregiver “…we can pick it up from [commercial pharmacy] but I didn't get an email about that. That's what we were waiting on because he only has a dose for tonight… if I need to, I can go pick it up for him at [hospital pharmacy]. Whatever is easier, just so he doesn't delay on that [antifungal].”

### 3.3 Outcomes Dimension: Impact on Patient Safety and Mental Health Outcomes

Participants discerned clear impacts of barriers to access and changes in care. One participant described intentionally taking lower doses of insulin to extend their supply (process change), adversely affecting their hemoglobin A1C level (outcome change).

Participant #42, Visit 1

Patient “I have a really hard time paying for the [insulin]. So, I was kind of skimping on it and the doctor wanted to know why was my A1C so high? I told him because I couldn't afford all that insulin. So, I just took … not as much as I needed to lower it.”

The psychosocial impact of insurance challenges was also clear through the patient‐clinician dialogue analyzed. Family caregivers described the excess time and travel required for these processes, which often increased caregiver strain. One family caregiver discussed the process of obtaining insurance approval for a follow‐up surgical procedure for the patient and its impact. This distress and frustration were equally challenging for the patient and family caregiver, who supported the navigation process and bore the consequences of the complex insurance landscape.

Participant #26, Visit 2

Psychologist “…you seem a little more discouraged than when I saw you before. Is that true?”

Patient spouse “Yeah …with trying to get insurance going, and they're [care team] supposed to be doing this thyroid surgery and I got that on my mind sometimes… and I guess I get discouraged.”

## 4 Discussion

This single‐center naturalistic study of patient‐provider dialogue highlighted ways in which health insurance changed care and shaped clinical assessment during ICU recovery clinic visits. We found that one in three participants raised health‐insurance‐related issues. In the real‐time context of health care delivery, patients, family caregivers, and clinicians alike identified the cost of care as it relates to out‐of‐pocket spending; the complexity of interfacing with health insurance companies; and health insurance literacy in the face of administrative burdens as structural barriers. Modifications to mediate health insurance barriers included patient‐initiated and cost‐related nonadherence to prescribed medications and treatments (e.g., reducing medications), as well as potentially unsafe “workarounds” to healthcare, posing a serious safety risk which could have devastating consequences for outcomes.

A fundamental role of insurers in modern US healthcare is to shape healthcare behavior. This may often take the form of driving toward lower cost medications, incentives for improved adherence to care recommendations, and reductions in low value care. Such efforts may be perceived as intrusions in clinician/patient interaction, yet they are still potentially net beneficial as they prevent inefficiencies from nontransparent pricing, principle‐agent incentive misalignment, cognitive limitations, time constraints, or idiosyncratic clinician preferences for nonevidence‐based recommendations. The impacts of lack of insurance on access to care are well known [15].

Our study highlights, however, multiple ways in which clinicians and patients may act with creative agency to attempt to subvert the spirit and letter of health insurance policies. In several cases, this results in behaviors that seem sensical within the framework of the clinical encounter yet potentially disastrous from a system perspective. Even in these physiologically brittle patients at high risk for readmission, key preventive medicines are being illegally imported from foreign nations without quality controls, and essential treatments for fungal disease are being skipped well before completion of therapy. This qualitative study complements multiple case reports in lay media, including those of patients replacing prescribed antibiotics with cheaper veterinary‐grade medications from online retailers [16].

Our results further suggest weaknesses of the typical appeals processes that many insurance companies have implemented—put in place precisely in recognition of the occasional poor fit between policies and specific cases. Such appeals require several characteristics that may be in short supply during ICU recovery. They require time without the denied therapy to be safe until the appeal is processed. They require time and significant energy, initiative, and cognitive skills to navigate the administrative burden of the appeal, all of which may be in short supply during the transition out of an ICU hospitalization [17, 18]. They often assume an established ongoing relationship with an outpatient clinician willing to lead efforts and supply documentation for the appeal—which many of these patients did not have and who, in any case, may not have sufficient familiarity with discharge documentation to efficiently process the appeal [19].

If these features of maladaptive responses to insurance policies—where the policies subvert their own goals rather than support them—are not uncommon, then a number of policy options should be considered. Presumptive eligibility for Medicaid has been a longstanding policy for children and those giving birth; it was identified as part of the tools for managing the COVID pandemic [20]. Expansion to critically ill patients would be possible. One could also envision expanded presumptive eligibility, potentially based upon length of ICU stay or severity of critical illness, that considers the job and income loss common after critical illness, rather than based only on pre‐illness status. However, as most of Medicaid's mandatory state benefits are focused on acute care, some of the most essential outpatient services for critical illness survivors fall under optional state benefits (e.g., physical therapy, occupational therapy, case management) and would remain uncovered by some states. Within managed care programs—both Medicaid and private—waiver of preauthorization for many medications and discharge needs might be of use (e.g., anticoagulant therapy, outpatient physical therapy) or their provision included in bundled payments for critical care recovery [4, 21]. Pilot programs and evaluations of such programs are needed, as follow‐up approaches for critical illness survivors have yet to be standardized.

This study has multiple strengths, including rigorous analysis of transcribed ICU recovery clinic appointments with healthcare clinicians. However, there are limitations. Our sample is from a single center in the United States, where the payor mix is below the US national average for private/self‐pay and Medicare/Managed Medicare categories and is almost double the US national average for Medicaid. Results may not be generalizable to other settings, particularly with regard to state‐to‐state differences in government insurance eligibility and coverage. As these were transcribed clinical care appointments, data were not collected systematically. Patients were routinely asked if the cost of medications is a problem; although this abbreviated approach initiated organic discussions regarding insurance coverage and may have provided richer insights that might not have been obtained via a controlled research forum, there is a chance that not all health insurance coverage insights were captured. Also, the study is embedded in a RCT which excluded patients with psychiatric disease, substance abuse, or acute neurological disease, a population that often is vulnerable to noncompliance and workarounds. Additionally, participants with connectivity barriers were excluded, potentially underrepresenting vulnerable populations known to be digitally disadvantaged (e.g., persons 65 years or older, lower socioeconomic status, persons with functional disabilities, those with other demographic disparities). Finally, we exclusively study individuals who had access to a post‐ICU recovery clinic. There is a considerable portion of the population without access to these services. Consequently, the actual situation may be worse.

## 5 Conclusions

Findings of this study provide initial insights into the barriers associated with obtaining and using US health insurance to access critical illness survivor care. These barriers may adversely influence affordable, timely, and appropriate follow‐up care. Future work should build on this investigation by examining the differences in insurance experiences for critical illness survivors across payor groups, which may assist in identifying potential multilevel solutions at the policy, payor, and healthcare system levels to mitigate these insurance barriers.

## Conflicts of Interest

The authors declare no conflicts of interest.

## Supporting information


**File S1.** Consolidated criteria for reporting qualitative studies (COREQ): 32‐item checklist.
