# Supplementary material for: Clinician and Patient Responses to US Health Insurers' Policies: A Qualitative Study of Higher Risk Patients
Source: Health Serv Res. 2025 Apr 24;60(3):e14615. doi: 10.1111/1475-6773.14615 (PMC12120516; doi:10.1111/1475-6773.14615)
Supplement: Supplementary file 1 — File S1. Consolidated criteria for reporting qualitative studies (COREQ): 32‐item checklist. [file HESR-60-0-s001.docx]

# **Supplementary File 1: Consolidated criteria for reporting qualitative studies (COREQ): 32-item checklist**

Developed from:

Tong A, Sainsbury P, Craig J. Consolidated criteria for reporting qualitative research

(COREQ): a 32-item checklist for interviews and focus groups. International Journal for

Quality in Health Care. 2007. Volume 19, Number 6: pp. 349 – 357

YOU MUST PROVIDE A RESPONSE FOR ALL ITEMS. ENTER N/A IF NOT APPLICABLE

| **No. Item** | **Guide questions/description** | **Section reported** |
| --- | --- | --- |
| **Domain 1: Research**  **team and reflexivity** |  |  |
| *Personal Characteristics* |  |  |
| 1. Interviewer/facilitator | Which author/s conducted the interview or focus  group? | Methods; refer to overarching study referenced in Methods |
| 1. Credentials | What were the researcher’s credentials? (e.g., PhD, MD) | Author list |
| 1. Occupation | What was their occupation at the time of the study? | Author list |
| 1. Gender | Was the researcher male or female? | N/A |
| 1. Experience and training | What experience or training did the researcher have? | Methods; refer to overarching study referenced in Methods |
| *Relationship with participants* |  |  |
| 1. Relationship established | Was a relationship established prior to study commencement? | Methods; refer to overarching study referenced in Methods |
| 1. Participant knowledge of the interviewer | What did the participants know about the  researcher? (e.g., personal goals, reasons for doing the research) | Methods; refer to overarching study referenced in Methods |
| 1. Interviewer characteristics | What characteristics were reported about the  interviewer/facilitator? (e.g., Bias, assumptions,  reasons and interests in the research topic) | Methods; refer to overarching study referenced in Methods |
| **Domain 2: Study design** |  |  |
| *Theoretical framework* |  |  |
| 1. Methodological orientation and theory | What methodological orientation was stated to  underpin the study? (e.g., grounded theory,  discourse analysis, ethnography, phenomenology,  content analysis) | Methods |
| *Participant selection* |  |  |
| 1. Sampling | How were participants selected? (e.g., purposive,  convenience, consecutive, snowball) | Methods; described in RCT publication |
| 1. Method of approach | How were participants approached? (e.g., face-to-face, telephone, mail, email) | Methods; described in RCT publication |
| 1. Sample size | How many participants were in the study? | Results |
| 1. Non-participation | How many people refused to participate or dropped out? Reasons? | Described in RCT publication |
| *Setting* |  |  |
| 1. Setting of data collection | Where was the data collected? (e.g., home, clinic, workplace) | Methods |
| 1. Presence of non-participants | Was anyone else present besides the participants  and researchers? | Methods; results |
| 1. Description of sample | What are the important characteristics of the sample? (e.g., demographic data, date) | Results |
| *Data Collection* |  |  |
| 1. Interview guide | Were questions, prompts, guides provided by the  authors? Was it pilot tested? | Methods; refer to overarching study referenced in Methods |
| 1. Repeat interviews | Were repeat interviews carried out? If yes, how  many? | Results |
| 1. Audio/visual recording | Did the research use audio or visual recording to  collect the data? | Methods |
| 1. Field notes | Were field notes made during and/or after the interview or focus group? | Methods; refer to overarching study referenced in Methods |
| 1. Duration | What was the duration of the interviews or focus  group? | refer to overarching study referenced in Methods |
| 1. Data saturation | Was data saturation discussed? | N/A QSA |
| 1. Transcripts returned | Were transcripts returned to participants for comment and/or correction? | refer to overarching study referenced in Methods |
| **Domain 3: Analysis and findings** |  |  |
| *Data analysis* |  |  |
| 1. Number of data coders | How many data coders coded the data? | Methods |
| 1. Description of the coding tree | Did authors provide a description of the coding tree? | Methods |
| 1. Derivation of themes | Were themes identified in advance or derived from the data? | Methods |
| 1. Software | What software, if applicable, was used to manage the data? | Methods |
| 1. Participant checking | Did participants provide feedback on the findings? | No |
| *Reporting* |  |  |
| 1. Quotations presented | Were participant quotations presented to  illustrate the themes/findings? Was each  quotation identified? (e.g., participant number) | Results |
| 1. Data and findings consistent | Was there consistency between the data  presented and the findings? | Results |
| 1. Clarity of major themes | Were major themes clearly presented in the  findings? | Results |
| 1. Clarity of minor themes | Is there a description of diverse cases or  discussion of minor themes | N/A |
